# Defining plant ecological specialists and generalists: Building a framework for identification and classification

**DOI:** 10.1002/ece3.9527

**Published:** 2022-11-24

**Authors:** Alex Kirsch, Matthew A. Kaproth

**Affiliations:** ^1^ Department of Biological Sciences Minnesota State University, Mankato Mankato Minnesota USA

**Keywords:** functional traits, generalist, metric‐based classification, oak, phylogenetics, Quercus, specialist, specialization

## Abstract

Specialization is a widespread but highly ambiguous and context‐dependent ecological concept. This quality makes comparisons across related studies difficult and makes associated terms such as “specialist” and “generalist” scientifically obscure. Here, we present a metric‐based framework to quantify specialization in 141 *Quercus* species using functional traits, biogeography, and species interactions. Rankings of specialization based on five metrics were used to answer questions about how specialization is used colloquially (i.e., individual species assessment by experts) and influenced by phylogenetics (Ancestral Character State Reconstruction, Automatic Shift Detection), biogeography (patterns of clustering by region and with climate), and species threat level (IUCN Red List). Metric‐based ranking can be representative of specialization in a consistent and practical manner, correlating with IUCN Red List data, and the mean scores of individual expert assessments. Specialization is shown to be highly correlated with precipitation seasonality and only moderately influenced by evolutionary history. Data‐deficient species were more likely to be highly specialized, and higher specialization was positively correlated with greater IUCN threat level. Frameworks for characterizing specialization and generalization can be done using metric ranking and can turn concepts that are often unclear into a definitive system. Metric‐based rankings of specialization can also be used to reveal interesting insights about a clade's evolutionary history and geographic distribution when paired with the related phylogenetic and geographic data. Metric‐based rankings can be applied to other systems and be a valuable tool for identifying species at risk and in need of conservation.

## Graphical abstract

A variety of metrics related to specialization are used to quantify and rank the concept in 141 species of Quercus (oaks). These rankings are used to explore evolutionary and biogeographic patterns, as well as specialization as a concept's usage by scientists.

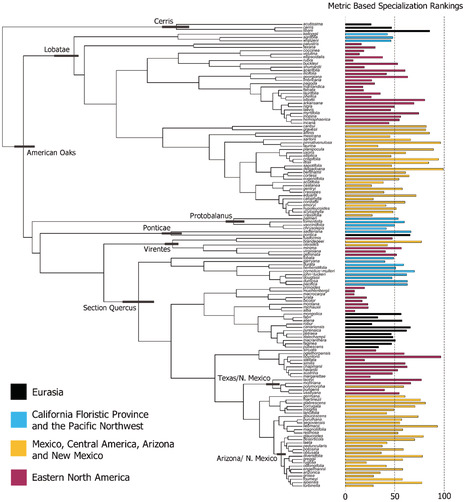

## 1 INTRODUCTION

Ecological specialization is a widespread concept in biological fields (Table 1; Berenbaum, 1996). This concept is valuable because specialist species have been shown to have unique characters and be at greater risk of extinction, making their identification critical to conservation efforts (Colles et al., 2009; Devictor et al., 2010; Dudley et al., 2019; Poisot et al., 2011). Some systems show no correlations between specialization and threat to a species, but this is often cited as a failure of characterizing specialization due to the complexity of the concept (Vázquez & Simberloff, 2001). Relevant terminology has become problematic due to ambiguity and variance, making comparison across studies difficult, and the ideas highly context dependent (Devictor et al., 2010). In certain contexts where specialization is clearly defined, it is often reduced to a single concept or metric, limiting designations to a single dimension (Aguilar‐Romero et al., 2017; Londero et al., 2017). Defining specialization more broadly can better represent a species' overall ecological strategy and may lead to better correlations with scientific opinion and species threat level, though limitations of comparisons between systems will still exist. Other studies have shown that standardizing terminology and definitions is of value (Avolio et al., 2019; Table 2).

**TABLE 1:** Short summary of factors related to specialization in literature concerned with ecological specialization

	Appearance of determining factors of specialization in literature			
	Habitat	Threat Level	Anatomical Features	Interspecies Interactions
Futuyma and Moreno (1988)	X	X	X	X
Marvier et al. (2004)	X	X		
Devictor et al. (2008)	X	X	X	
Poisot et al. (2011)	X		X	X
Sverdrup‐Thygeson et al. (2017)	X	X	X	
Londero et al. (2017)		X		
Ramiadantsoa et al. (2018)	X	X		X
Reed and Tosh (2019)	X	X	X	
Zettlemoyer et al. (2019)	X	X	X	

**TABLE 2:** Glossary

Unambiguous specialist	Species with one or more of; restricted range/low tolerance in habitat diversity, anatomy with narrow usage, high reliance on or evolution against interspecies interactions, are highly threatened
Ambiguous specialist	Species that express some level of the same qualities as unambiguous specialists, but at a more intermediate level, making their designation as specialized subjective
Generalist	Species with one or more of; large relative range of tolerable habitats, high phenotypic plasticity, varied resource usage, high tolerance to disturbance/ability to capitalize on disturbance
Ecoregion	A major ecosystem defined by distinctive geography, that receives uniform solar radiation and moisture
IUCN designation	How threatened a species is, as evaluated by the IUCN Red List of species. I.e., “Critically Endangered”, “Least Concern”, “Near Threatened”
Biological level	Taxonomic rank being considered. I.e., species to species, genus to genus, order to order

Attempts to define and classify specialization are numerous and add to the increasing number of definitions assigned to the concept (Ferry‐Graham et al., 2002). Studies also often do not make the biological level being referenced clear. Species may be considered specialists relative to their clade, but generalists compared to other genera (Devictor et al., 2010). Issues are further compounded by the fact that many of these studies are purely theoretical and do not attempt to apply concepts to a specific system. This leads to questions such as do experts assess generalized and specialized species in a consistent manner? If they do, can we infer which traits' experts are utilizing to make these designations? The factors that are cited as determining specialization and generalization are often consistent across related literature when they are mentioned (Table 1).These factors are often clear to see when looking at *unambiguously specialist* species (Table 2).

Consider family *Droseraceae* (sundews; Gonella et al., 2016). Members are carnivorous, herbaceous plants that are endemic to bog‐like conditions, and their specialized traits grant them robust fitness in these nutrient‐poor, acidic environments (Thum, 1989). *Drosera* spp. leaves are also unambiguously specialized, having been modified into an adhesive snare that immobilizes prey for digestion (Thum, 1989). Unambiguous specialists such as these are valuable for characterizing specialization, as their extreme state across multiple characters makes what factors influence the designation clear; narrow habitat preference, highly modified anatomy with potentially narrow use, and a high dependence on other members of the same ecological network. They also demonstrate how a species can become restricted to its evolutionary peak, becoming so specialized that evolution into new environmental niches becomes difficult (Wright, 1932).

Problems arise when assigning specialist and generalist designations without making the biological context clear, and when considering species that are *not* blatantly specialized. Ambiguous specialists are exactly that; species that could be considered either a specialist or generalist, depending on how you frame your justification (Table 2). Consider *Asarum canadense* (wild ginger), which occupies many kinds of upland forests but generally never occurs in habitats that would not be considered forests. Is wild ginger a specialist, highly evolved for upland forests, or a generalist that can inhabit multiple habitats with forest conditions? We cannot say without context. This species may have a narrow set of ecological conditions to utilize *relative to its clade*, but might seem quite far reaching compared to *other genera*. In reality, it could be both, and it is more pragmatic to consider specialization and generalization as a spectrum where most species fall somewhere in the middle.

We propose a system for characterizing specialization within a clade, where specialization and generalization form a spectrum, rather than act as binary designations, sensu Ainsworth and Drake (2020). We demonstrate the system's utility by examining ecological specialization in the *Quercus* clade, allowing us to answer questions surrounding specialization: (1) Does colloquial usage of generalists and specialists correlate to measurable traits? (2) Are specialists more threatened? (3) What kind of emergence patterns does specialization follow? We hypothesize a pattern similar to that proposed by Holt (2009) in which populations of generalists give rise to specialized descendants once they have expanded into new territories and speciated.

### 1.1 Comparisons to Grime's CSR triangle

One system of classifying ecological strategies is Grime's Competition‐Stress‐Ruderal triangle (Grime, 1977). Each axis of the triangle represents an area where species are forced into tradeoffs, and species that fall near the tips are exhibiting the extreme form of a strategy. Calculating ecological strategies using functional leaf trait data in conjunction with the CSR framework has been shown to be both feasible and of value to related fields, even at a global scale (Pierce et al., 2017). The spectrum of strategies formed by Grime's triangle has some similarities to the spectrum formed between specialization and generalization. Species can be placed on a CSR triangle surface relative to others to estimate how adapted they are for certain strategies. The area a species covers would convey how specialized their traits are to facilitate a strategy (sensu Pierce et al., 2017).

The main problems with using the CSR framework to categorize specialization are threefold; (1) the categories do not directly translate into a linear continuum of specialization to generalization, (2) species may differ in ecological strategy but still be placed on the same region of the CSR triangle, and (3) multidimensionality makes analyses impractical. Co‐occurring species in a high‐stress environment may not share the same ecological strategy—one may be specialized to conserve captured resources while the other may be a generalist with high plasticity (Emery et al., 1994). It may be that rather than being directly comparable, specialization into a given habitat drives ecological strategy (Dayrell et al., 2018), where tradeoffs are a direct consequence of adaptive specialization (Agrawal, 2019). On a CSR triangle, a specialist's ecological niche should be narrow (a point) while a generalist would have a range of breadth (with measurable area). Furthermore, by conscribing a species by its plasticity and using several metrics to compare to sister species (within a clade), we present a framework that can capture CSR area questions and reduce them to one dimension. While position on the CSR triangle is a three‐dimensional metric, our system produces one‐dimensional rankings, enabling practical comparative systematics investigations when coupled with phylogenetic data.

### 1.2 Study system

To demonstrate how a species' degree of specialization can be characterized, genus *Quercus* is utilized as an ideal system to study specialization (Cavender‐Bares, 2019). *Quercus* is comprised of approximately 455 species of trees and shrubs (Nixon, 1993), with some boasting a wide distribution while others are found only in very narrow ranges (Cavender‐Bares et al., 2018; Hauser et al., 2017; Kremer & Hipp, 2019; Manos, 2020; Nixon, 2002). Species inhabit regions of extreme climates (drought and high‐water availability, and areas with mild to severe winters) and soils (Hipp et al., 2018). *Quercus* species also express a high degree of functional leaf trait plasticity, with plasticity in leaf traits showing coordination between one another as a response climate (Ramírez‐Valiente et al., 2020).

Variation among *Quercus* spp. continues when considering their breadth of interspecies relationships. Species have documented interactions with a wide array of herbivores, and as a result produce a diverse arsenal of defensive traits (Moreira & Abdala‐Roberts, 2020; Pearse & Hipp, 2012). This large variation in habitat preference and physiology, as well as the bulk of existing species, means that this genus likely contains species of varying degrees of specialization. Without differentiation, it is unlikely that *Quercus* would be so widespread and prominent, being ecologically dominant in North America and spanning the northern hemisphere (Kremer & Hipp, 2019). With a high biological relevance, research done on *Quercus* species is valuable to conservation efforts (Carrero et al., 2020). Specialists are thought to be more highly threatened by disturbance and anthropogenic effects, although this is at times disputed (Colles et al., 2009; Monks & Burrows, 2014). More notable, however, is that data related to these species are abundant, readily available, and diverse (Kaproth & Cavender‐Bares, 2016; Moreira & Abdala‐Roberts, 2020; Pearse & Hipp, 2012).

## 2 METHODS

### 2.1 Ranking process

**FIGURE 1:**
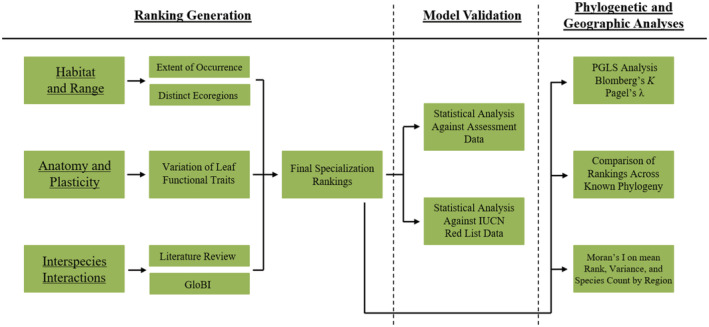
Flowchart of overall methodology. A priori ranking generation was built on five metrics (later reduced to four due to the model selection process dropping Domatia presence). The leftmost column contains concepts identified as being relevant to specialization in literature, with the next column to the right representing the metrics we used to represent those concepts. Final specialization ranking metrics (determined through model selection) were validated against two control datasets (survey of experts and IUCN red list data). Final specialization rankings were then used to explore phylogenetic and geographic patterns of development and adaptation.

To objectively assess specialization, we developed a quantitative ranking system comprised of five metrics (Table 3) similar to trait‐based approaches used by Morelli et al. (2019) and Ainsworth and Drake (2020). Traits were chosen through a combination of a priori research and AICc model selection. Species were assigned points toward specialization based on value in each metric compared to all other *Quercus* species in this study, and scores for each metric were summed to create the final metric‐based specialization ranking (*Ranking Generation*; Figure 1). Not all collected metrics were utilized in this process, some having been omitted due to the model selection process outlined below. Rankings were produced for 141 *Quercus* species, a large subset of the species included in the maximum likelihood phylogeny of the American oak clade generated in Hipp et al. (2018). Similar metric‐based systems could be utilized for most clade level investigations. Rankings were tested against IUCN Red List data (IUCN, 2021) to look for correlation between specialization and threat level. Rankings were similarly compared to the results of a specialization survey, where experts familiar with *Quercus* were asked to rank the specialization of species (*Model Validation*; Figure 1).

**TABLE 3:** Ranking metrics, how their relationship to specialization–generalization is interpreted, and the source of the related data

Metric	Interpretation for specialization	Data source (and method)
Extent of Occurrence (EOO)	More specialized species should have a smaller EOO (specialists have smaller ranges; IUCN, 2012)	Hipp et al., 2018 (ArcMap)
Number of Distinct Inhabited Ecoregions (DE)	More specialized species should inhabit less distinct ecoregions (specialists have more restricted habitats; Caley & Munday, 2003)	Generated using Hipp et al. (2018) samples with Level III ecoregions of North America (ArcMap)
Trait Plasticity (Petiole Length, Leaf Length, Leaf Lobedness, Specific Leaf Area, Perimeter per unit Leaf Area, Leaf Venation)	More specialized species should be less plastic (lower plasticity is associated with a higher vulnerability to disturbance; Murren et al., 2015)	Calculated from Kaproth et al., 2020 (Supporting Information, Formula 2)
Number of Notable Documented Interspecies Interactions	More specialized species should have a higher number of notable species interactions (specialized species are thought to rely on interspecies relationships; McCann, 2007)	Global Biotic Interactions Tool 2021 (GloBI)
Domatia Presence	Having leaf domatia is interpreted as making a species more specialized (domatia represents specialized anatomy in oaks; Shenoy & Borges, 2010)	Digitized herbaria (Kaproth et al., 2020, SEINet, Oaks of the World, and iNaturalist)

*Note*: All traits below other than those underlined (Domatia presence, leaf venation, and perimeter per unit leaf area) were included in the final AICc selected metric‐based rankings model.

### 2.2 Metric‐based specialization rankings and percentile scoring

Metric‐based specialization rankings are numeric values with higher values representing higher specialization, and lower values representing higher generalization. Depending on where a species' metric value falls within the range for all species for that metric, it is assigned points toward its final specialization ranking. For example, species with small ranges relative to other *Quercus* members get more points toward specialization. This was repeated for every metric for 141 species. The totals of a species' four metric scores were combined to produce its final ranking, with each representing 25% of the total. Domatia presence would have been the fifth metric, making each represents 20% of the total, but was dropped from the model due to the process outlined in the following section. Species that were data deficient had the weighting of their available metrics adjusted to compensate. For example, a species with three of the four metrics available would have each metric make up 33% of the total instead. This was done to keep data‐deficient species on the same scale as fully represented species. How each metric was obtained, the reasoning for its inclusion, and its associated calculations are outlined below.

### 2.3 Model selection

To independently generate traits for metric‐based specialization rankings, an initial literature review and a priori metric selection process were utilized. This yielded more metrics than those included in the final rankings of specialization (Table 3). Akaike information criterion (AICc) was used to compare models for predicting both IUCN Red List designation and average expert survey score. Metrics that appeared in one of the most optimized predictive models for these two datasets were used in ranking generation. All gathered metrics were utilized except for plasticity of perimeter per unit leaf area, plasticity of Leaf Venation, and Domatia presence (Table 3; Supporting Information‐Part 1). The resulting model has metrics that represent ecological, physiological, and geographical data. A stronger predictive model with an ~3% lower AICc value is possible, but this model contains only plasticity metrics (plasticity of leaf lobedness, specific leaf area, petiole length) and is no longer representative of overall specialization. Without the inclusion of varied data, we lose the ability to make inferences about patterns in specialization and scientific assessment of the concept. These results were validated through phylogenetic generalized least squares (PGLS) models in R version 4.0.4 (*R* Core Development Team, 2019) with packages APE v.5.4.1 (Paradis & Schliep, 2018), MAGRITTR v.2.0.1 (Bache & Wickham, 2022), NLME v.3.1.152 (Pinheiro et al., 2020), and PHYTOOLS v.0.7.80 (Revell, 2012) in comparison with AICc selection outputs from JMP (Version 15.1.0). The phylogeny utilized for the PGLS analyses was pruned from Hipp et al. (2018), with data‐deficient species being dropped from the tree. The resulting phylogeny had 91 *Quercus* species at the tips.

### 2.4 Specialization survey

To create a comparative dataset of *Quercus* specialization, experts familiar with *Quercus* species were asked to rank species on level of specialization, and to define specialization and generalization (Supporting Information‐Part 1, Survey Sample). Metric‐based data were compared to these results to gain insights into consistency of specialization evaluation from experts. These results were also analyzed to determine what factors experts may have been using in their designations of specialization. Surveys were sent via email in both Spanish and English to 42 respondents across three regions to which the relevant *Quercus* species are native. Twenty‐six respondents completed the survey for an average of 3.8 responses per species. Survey respondents showed a variety of backgrounds and occupations, with many coming from academia, arboretums, herbariums, and other groups that work closely with *Quercus* species.

### 2.5 Extent of occurrence

**FIGURE 2:**
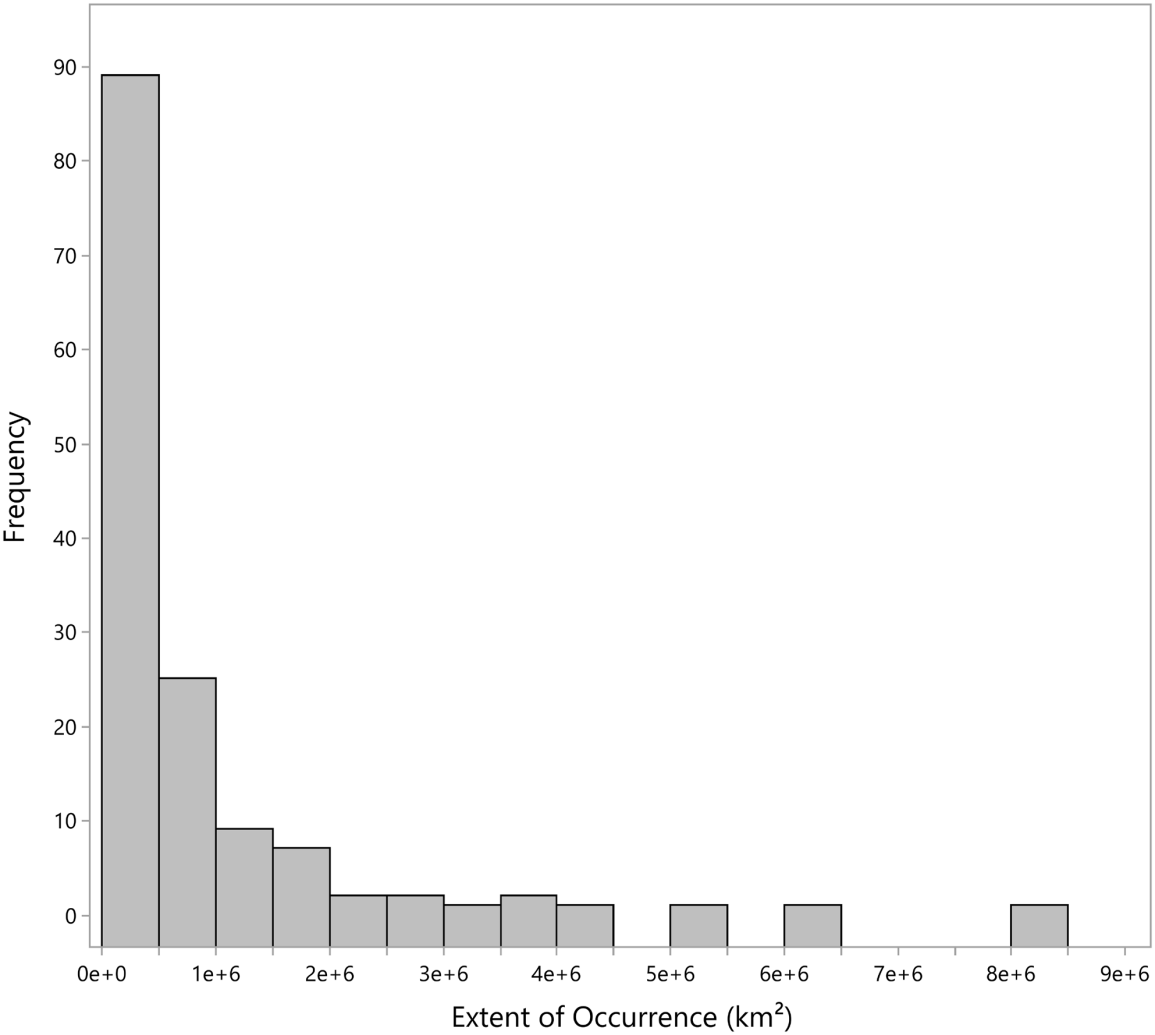
Histogram of extent of occurrence (EOO) of 141 Quercus species. 92.2% of these species have an extent of occurrence below 2,000,000 km^2^.

A species' extent of occurrence (EOO) is defined as the area contained within the shortest continuous imaginary boundary that can be drawn to encompass all the known and inferred sites of present occurrence of a taxon, excluding obvious cases of vagrancy (Guidelines for assessing the conservation status of native species, Environment and Biodiversity Conservation Act of 1999). EOO was calculated from 150,886 georeferenced *Quercus* specimens (Hipp et al., 2018). Values were calculated using ArcMap v10.8.1 following the guidelines set forth by the Threatened Species Scientific Committee (a diagram is provided in the Supporting Information‐Part 1, Figure S1). The top 20th percentile species were assessed as having max EOO scoring due to the logarithmic distribution of values (Figure 2). These 29 species were assigned zero points toward specialization from EOO; the remaining scores were calculated using Formula 1 (Supporting Information‐Part 1).

### 2.6 Number of distinct inhabited ecoregions

Ecoregions are geographic areas where ecosystems and environmental resources are generally similar (Omernik, 1987). Ecoregions come at different levels of resolution, with higher levels having more subdivisions. Level I divides North America into 15 ecoregions, while level III defines 182. Central and South America, as well as the Caribbean, are broken into 12 regions at level I, and 121 regions at level III. Level III is used throughout this study, as the level of detail is the highest without imbalance between regions. At lower levels, many *Quercus* species with greatly differing ranges would only inhabit one ecoregion, making it nonfunctional as a differentiating metric. Methods used to define ecoregions are given in Omernik (1995, 2004) and Omernik and Griffith (2014). Here, the number of ecoregions a species occurs is used as a measure of niche breadth and a species' ability to utilize a variety of resources and conditions.

The Hipp et al. (2018) dataset includes 877 ± 2385 (SD) unique presence records per species for each of 137 species. Thirteen species had 10 or fewer records, while 98 species had at least 50 occurrences. ArcMap was used to map level III ecoregions with presence records overlain to produce a dataset of what ecoregion each sample occurred in. Processing in R produced a count of distinct ecoregions inhabited per species (DEL3, distinct ecoregions at level III). Inhabiting a lower number of ecoregions was interpreted as meaning a species is more specialized. Scoring for DEL3 also done using Formula 1 (Supporting Information‐Part 1). DEL3 ranged from a high of 59 distinct regions (*Q*. *rubra*) to a low of 1 (10 species).

### 2.7 Plasticity

Trait plasticity is thought to be a useful quality for generalist species to adapt and persist in varied environments—meanwhile, specialized species may lack plasticity at both an individual and evolutionary level (Griffith & Sultan, 2012; Marvier et al., 2004). The development of plasticity is also thought to be critical for species range expansion and allowing a species to persist in a niche when environmental and ecological disturbances arise (Palacio‐López et al., 2015; Valladares et al., 2014). Here, we characterized oak leaf plasticity through novel use of herbarium data to investigate patterns in a large clade (sensu Heberling, 2022). We represent plasticity as variation in four functional leaf traits (petiole length, leaf length, leaf lobedness, specific leaf area; sensu Cornelissen et al., 2003), calculated from specimens provided in Kaproth et al. (2020) with Formula 2 (Supporting Information–Part 1). Species had an average of 14 ± 12 (SD) individuals, with 115 of 136 species having more than three specimens. Herbarium specimens were standardized to minimize acclimation differences (all leaves were sun grown, and shade grown leaves were avoided). To partially control for variation that may be abnormal for a species (e.g., hybrids), the functional trait herbarium specimens were validated against oaks grown ex situ (in common gardens and arboretums) and in situ (field observations by experts).

The four functional leaf traits were chosen due to their correlations with leaf economics, hydrologic niche, and/or ecophysiological performance in angiosperms (Ramírez‐Valiente et al., 2020). Petiole length has been shown to influence light capture per unit leaf area (Takenaka, 1994). Leaf length is known to respond to drought and be influenced by environmental pressures (Barre et al., 2015; Deblonde & Ledent, 2001). Leaf lobedness shows the same patterns as leaf length, but additionally has also been shown to be critical as a means to control hydraulic resistance and water balance (Baker‐Brosh & Peet, 1997; Sisó et al., 2001). Specific leaf area is tied to ecological niche and highly correlates to foliar nutrient content, which has used as an indicator of plant response to disturbance (Firn et al., 2019; Hoffmann et al., 2005; Ramírez‐Valiente et al., 2020).

### 2.8 Interspecies interactions

Many species globally are specialized by virtue of ecological and evolutionary relationships with other species, including both mutualists and symbiotes as well as species highly adapted to defend against other species. Here, specialization via interspecies interactions is represented by the presence and quantity of known interactions on the Global Biotic Interactions tool (Supporting Information–Part 1).

### 2.9 Domatia

Anatomical features with narrow utility are a notable aspect of some specialized species. Although *Quercus* is considered largely generalist in a broader sense, domatia represents specialized anatomy that can be assessed for the oaks. In *Quercus*, domatia are small chambers made of trichomes at the intersections along the mid‐vein of the leaf. These are created to shelter beneficial arthropods that likely help reduce herbivory on the tree. The presence or absence of domatia may be interpreted as being indicative of interspecies specialization.

Domatia presence or absence was assessed for three individuals per species. Each of the three samples was denoted with a 0 (no domatia), 1 (hair present but likely nonfunctional), or 2 (functional domatia present). These were summed per species, and the totals were scored using Formula 1 (Supporting Information‐Part 1), but without inverting scores, as higher domatia presence was interpreted as higher specialization.

### 2.10 Model validation and testing consistency of ecological concepts

**FIGURE 3:**
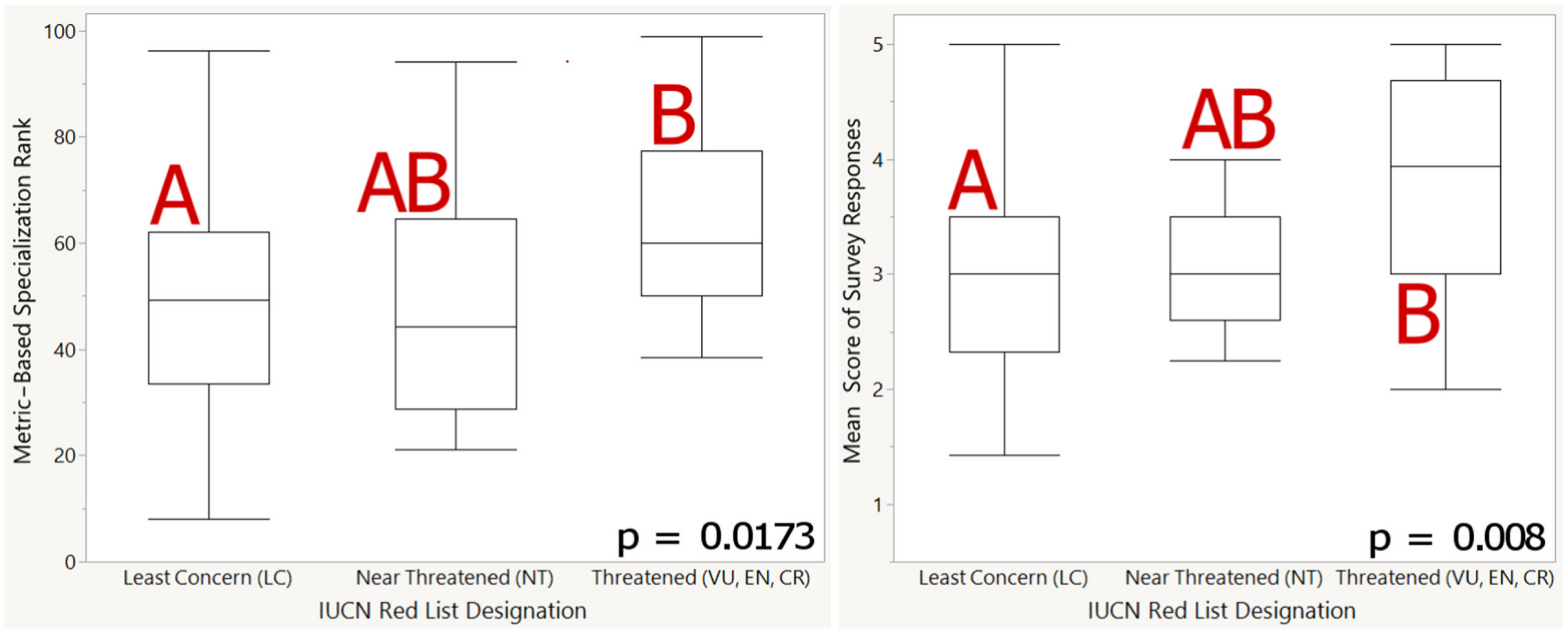
Alignment of metric‐based specialization rankings and expert survey score with IUCN red list designations. Tukey–Kramer connecting letters denote differences.

To validate the model, associations among the three models of species designation were tested (Figure 3): Metric‐based specialization rankings and IUCN designations, specialization rankings and survey results, and survey results and IUCN designations. This also allowed for the detection of any potential inconsistencies between metric‐based rankings, scientific discourse, and conservation efforts.

### 2.11 Phylogenetic methods

To investigate the relationship between evolutionary history and specialization, metrics related to the concept of specialization were tested across the *Quercus* phylogeny for Blomberg's K (to analyze differences in specialization across clades) and Pagel's *λ* (to test covariance across the tips of the phylogeny). The metrics utilized were extent of occurrence (EOO), number of distinct inhabited ecoregions at ecoregion level III (DEL3), plasticity of four functional leaf traits, and the number of notable documented interspecies interactions (Table 3). Tests for phylogenetic signal were performed using the R package PHYLOSIGNAL v.1.3 (Keck et al., 2016). R was used to generate all phylogenetic figures (Supporting Information‐Part 2). An Ancestral Character State Reconstruction was also performed using the ape package, to infer ancestral conditions of specialization using our metric‐based specialization rankings for modern species, and to further explore evolutionary trends (Irisarri & Zardoya, 2013; Figure 5). We also tested for phylogenetic shifts in metric‐based specialization rankings and individual ranking metrics by evaluating the relative support for alternative Ornstein–Uhlenbeck (O–U) models. This process models transitions in trait values as responses to shifting selective regimes (Bastide et al., 2018; McCormack et al., 2020). The analysis was performed using an Expectation Maximization (EM) search algorithm (Bastide et al., 2018) over the space of 10 transitions, with the O–U model for independent traits. Phylogenetic shift testing was performed using the R package PHYLOGENETICEM v.1.4.0 (Bastide et al., 2017).

### 2.12 Geographic methods

Clustering and variance of specialization by ecoregion and the number of distinct species per ecoregion were plotted in ArcMap (10.8.1) and analyzed using a Moran's *I* (Figures 6, 7). Mean metric‐based specialization ranking in each region was determined as the mean specialization value of distinct species that appeared in said region. To assess environmental influence on generalization, PGLS analyses were also performed between metric‐based specialization rankings and mean species environmental traits for precipitation seasonality (Bioclimatic variable 15; Hijmans et al., 2005; Figure 8). This climate factor was chosen due to its prominence in explaining ecological adaptation patterns in prior system work, as well as the relative importance water availability plays as a selection pressure (sensu Cavender‐Bares et al., 2018; Cavender‐Bares, 2019; McCormack et al., 2020). This analysis was performed using the generalized least squares (GLS) model of the NLME package in *R*, using a maximum likelihood method.

## 3 RESULTS

### 3.1 Correlations between metric‐based specialization rankings, specialization survey results, and IUCN red list designations

Metric‐based specialization rankings, specialization survey data, and IUCN Red List designations all significantly and positively correlate with one another. A paired *t‐test* between survey data and ranking data reveals that while the two datasets correlate, experts tend to rank species about 14% higher, or more specialized on average (*p* > *t* = <.0001, Mean Difference = 13.6, *t*‐Ratio = 7.54). Expert survey responses aligned most with extent of occurrence (*p* = .0005, *R* ^2^ = .10). Survey data and IUCN designations tested significantly with a one‐way ANOVA (*p* = .0089), and a Tukey–Kramer connecting letters report revealed that while species of least concern (LC) differed significantly from those that were threatened (i.e., any designation more severe than Near Threatened, NT), species that were near threatened could not be determined to significantly differ from either the species of least concern or those that were threatened. Compared to species of least concern, experts scored near threatened species as 1% less specialized on average, and threatened species 29.1% more specialized on average. When comparing metric‐based specialization rankings and IUCN data, the relationship was also significant, and the connecting letters report exhibited the same pattern (*p* = .0373); these results are shown graphically in Supporting Information‐Part 1, Figures S4,S5. When compared to species of least concern, near threatened species were ranked as 3.3% more specialized on average, and threatened species were ranked 35.5% more specialized on average. While there was some overlap between the metrics utilized by the IUCN Red Listing process and the metric‐based specialization rankings, namely in extent of occurrence, AICc model selection revealed that overlapping metrics were not favored as predictive variables.

### 3.2 Phylogenetic signals and plasticity correlations

Tests for phylogenetic signal (Blomberg's K and Pagel's *λ*) yielded significant models for the Metric‐based specialization rankings and four of the five metrics used to build the rankings (Table 4). Values of K indicated variance between species is within clades, rather than among them. Values of *λ* for metric‐based specialization rankings, extent of occurrence, total domatia score, and total plasticity indicate low to moderate phylogenetic signal, meaning the tested metrics have evolved with less phylogenetic influence than a Brownian Motion (BM) model would predict. DEL3 showed a high *λ*, which is indicative of trait evolution according to what a BM model would predict. Number of interspecies interactions did not indicate a phylogenetic signal. Plasticity traits tend to correlate highly with other plasticity traits (Supporting Information‐Part 1, Figure S2), or in other words, species that are plastic for one leaf trait are usually plastic for many traits.

**TABLE 4:** Phylogenetic signal of metric‐based specialization ranks and factors of specialization individually (Blomberg's *K* and Pagel's *λ*)

Character	*K*	*p*	*λ*	*p*
Metric‐based specialization rank	0.132514	.013*	.472912	<.001*
Extent of occurrence	0.14894	.070	.412588	<.001*
Distinct ecoregions at level III	0.204776	.001*	.832833	<.001*
Total Domatia score	0.129575	.017*	.421461	<.001*
Number of interspecies interactions	0.0675066	.962	.000067	1
Total plasticity	0.175	.003*	.279	.002*

*Note*: Total plasticity is the sum of plasticity in the six traits that were measured for plasticity (values for individual leaf traits are in their own table in the Supporting Information‐Part 1, Table 1). Asterisks* denote significance. Unexpected phylogenetic shifts were only detected for species extent of occurrence (Supporting Information‐Part 1, Figure S3).

### 3.3 Metric‐based specialization rankings, the Quercus phylogeny, and native regions

**FIGURE 4:**
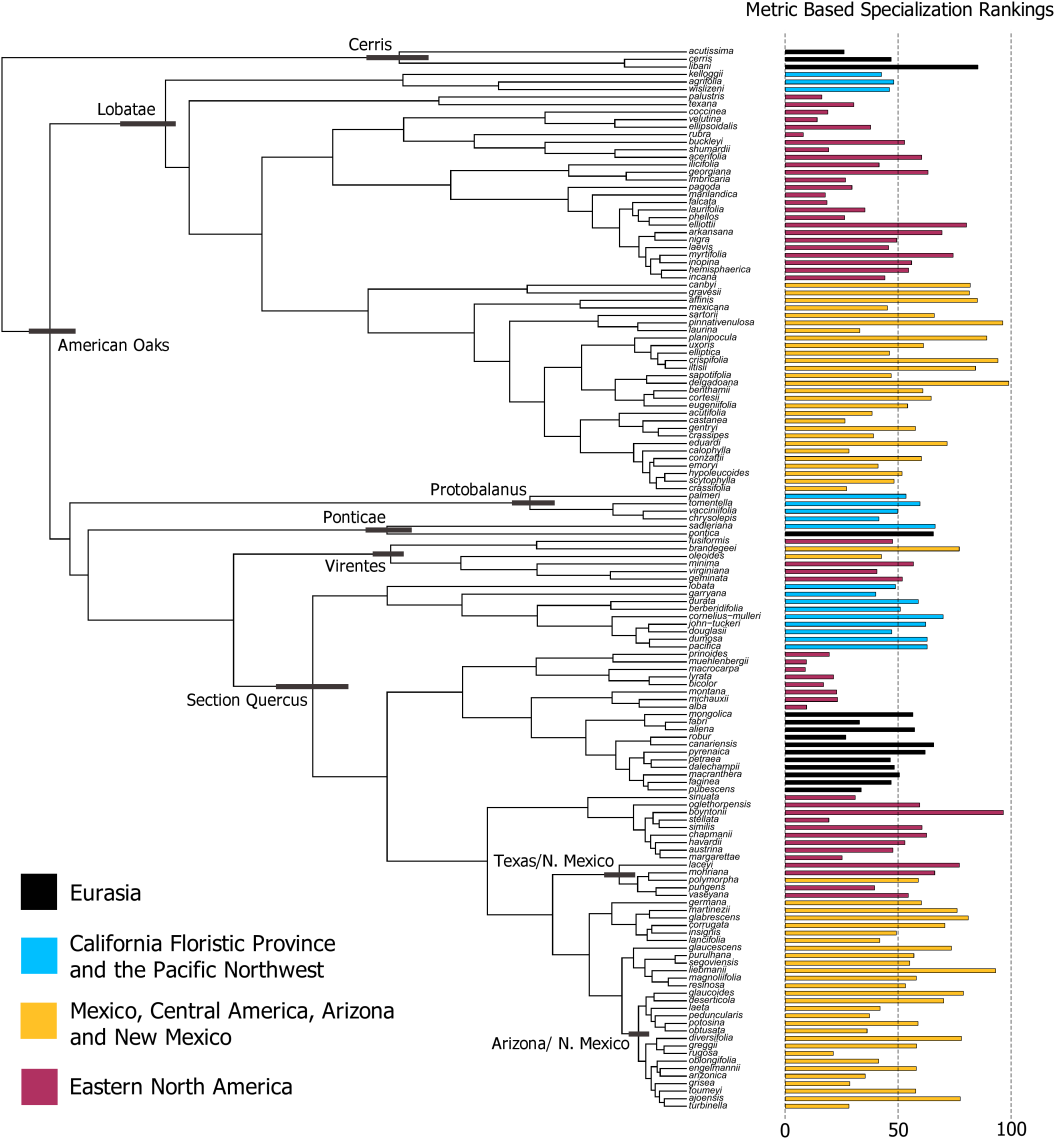
Specialization rank and region of *Quercus* species across *Quercus* phylogeny (141 species). Major groups are defined at their respective nodes, and the length of the gray bars indicates the relative uncertainty in dating (sensu Hipp et al., 2018). Longer bars at the tips of the tree represent higher specialization (above 50), while short bars indicate more generalized species (below 50). Color of the bar corresponds to a species' native region.

Within the *Quercus* phylogeny, there are visible patterns with respect to species metric‐based specialization rankings and their native regions (Figure 4). The most striking pattern is exhibited by the Eastern North American (ENA) clade within Section *Quercus* spanning *Q*. *prinoides* to *Q*. *alba* (following descriptions provided by Manos & Hipp, 2021). This clade contains some of the most generalized species in the study, with rankings ranging from 8.9 (*Q*. *macrocarpa*) to 23.0 (*Q*. *michauxii*), and a mean ranking of 16.2 ± 2.2. Sister taxa of this clade inhabiting the California Floristic Province and Pacific Northwest (CFPN) show a slight bias toward specialization, with a mean ranking of 55.9 ± 3.2. Sister taxa native to Eurasia lack a clear bias toward specialization or generalization, with a mean ranking of 47.8 ± 3.8. Overall, rankings of natives of the CFPN and Eurasia tend toward the midpoint of 50. Of the 104 species with all metrics available, *Q*. *myrtifolia* scored the highest, at 74.3, and *Q*. *rubra* scored the lowest, at 8.0.

**FIGURE 5:**
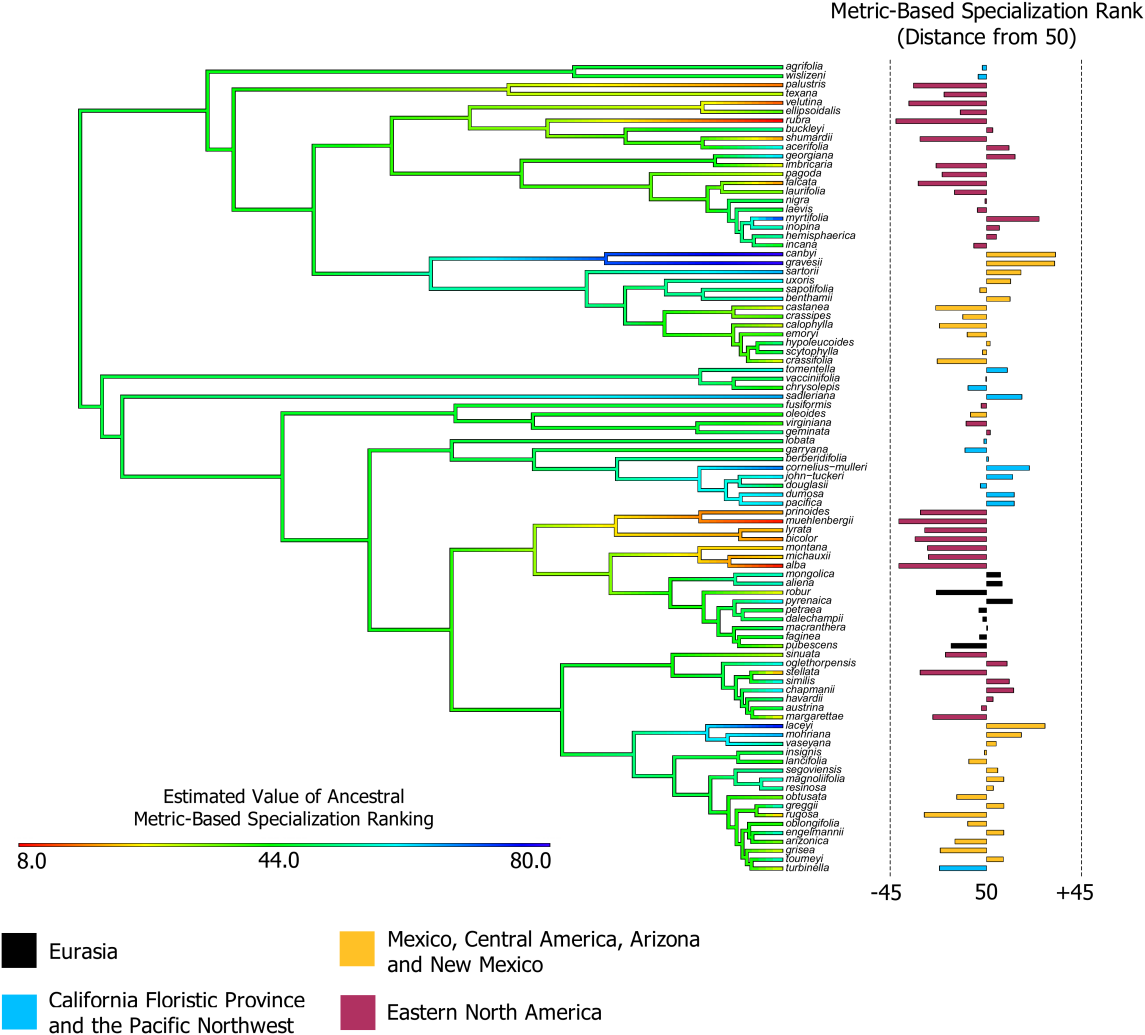
Ancestral character state reconstruction of metric‐based specialization rank across *Quercus* phylogeny (91 species). Bars at the tips represent metric‐based specialization rank as a distance from 50 (the midpoint of possible rankings). Bars that extend right are species ranked as specialized, while bars extending left are more generalized; bars are color coded by region. The branches of the tree are color coded by the estimated metric‐based specialization ranking of ancestors.

**FIGURE 6:**
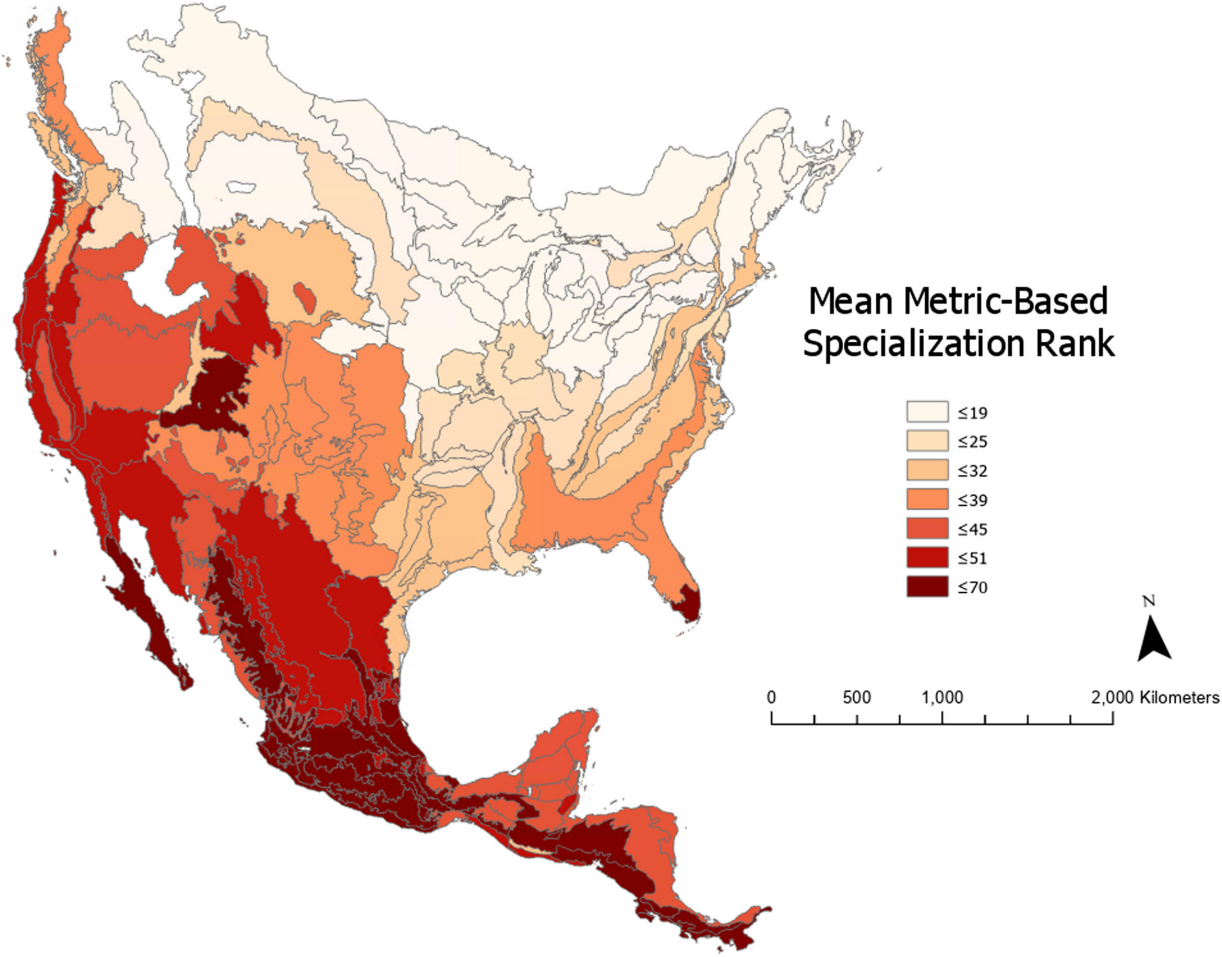
Mean metric‐based specialization rank of species within each ecoregion across the continental United States through Mexico and Central America.

**FIGURE 7:**
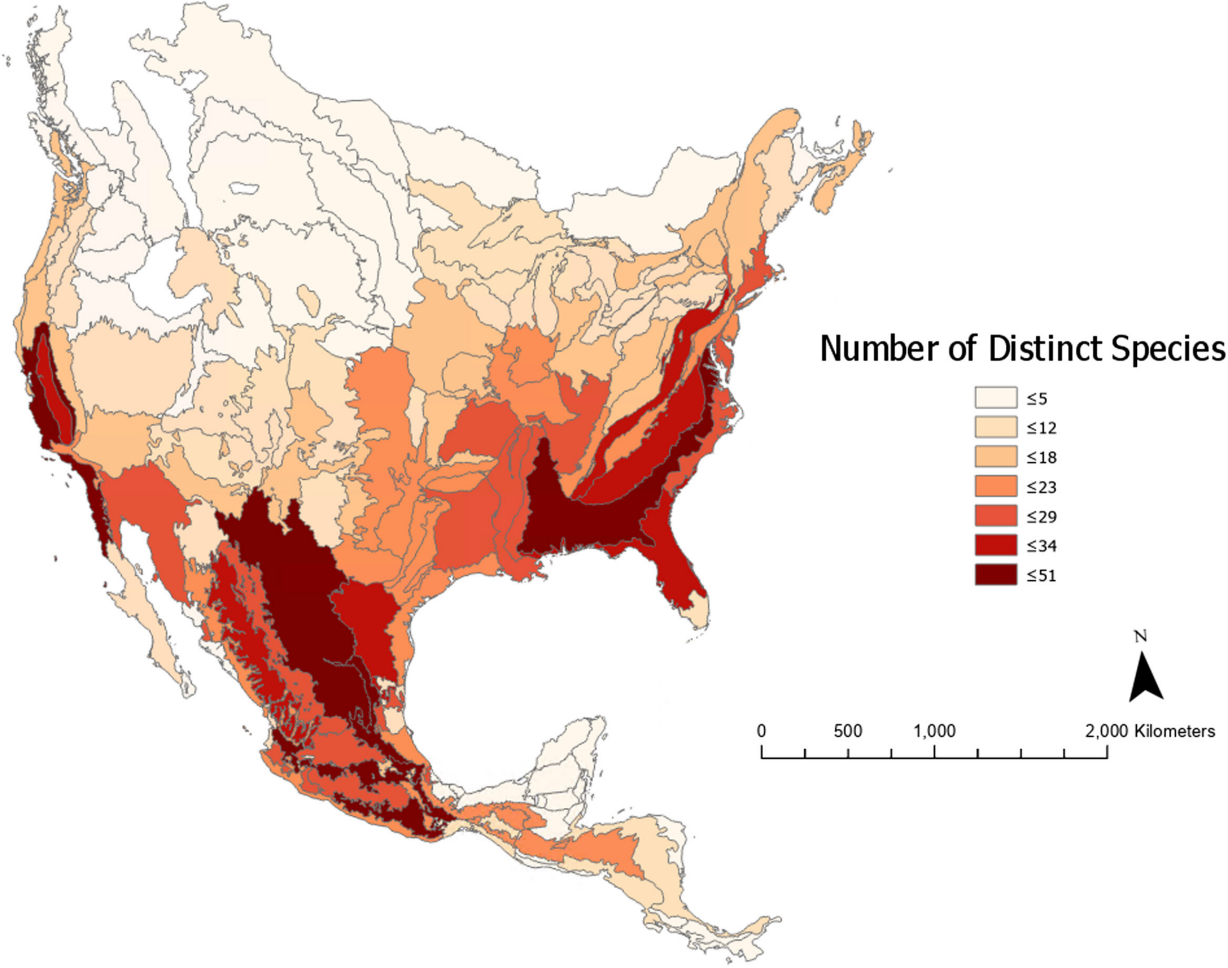
The number of distinct species inhabiting the ecoregions of north and Central America. *Quercus* species diversity is highest in the Southeast United States, the United States west coast, and large ranges in Mexico; the number of distinct species is significantly clustered (Moran's *I*, *p* < .0001; Figure 7). Species diversity tends to be higher in transitional regions that exist between specialist and generalist dominated regions. These likely contain areas that can accommodate both strategies (e.g., Southeastern Plains). This region spans states bordering the gulf such as Mississippi, Alabama, and Florida, up through much of the US east coast to states like Virginia and North Carolina. Ecoregions in the intermountain west show low species diversity, with most ecoregions containing less than 12 distinct species.

**FIGURE 8:**
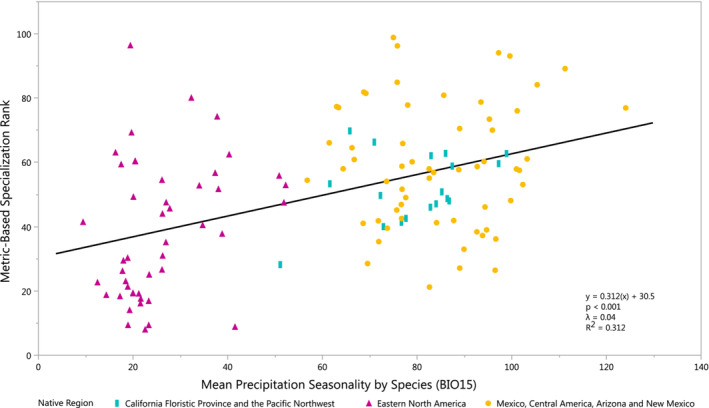
Metric‐based specialization is correlated to Quercus species mean precipitation seasonality (bioclimatic variable 15).

ENA natives that are more closely related to natives of Mexico, Central America, Arizona, and New Mexico (MCAN) are more prone to higher specialization (*Q*. *elliottii* 80.1, *Q*. *boyntonii* 96.5, *Q*. *laceyi* 77.1). MCAN species show the greatest propensity for specialization, with a mean ranking of 58.7 ± 2.6. Even the most highly generalized representative from this group, *Q*. *rugosa*, scored 21.2; over twice as high as some of the most generalized representatives of ENA. Despite this, the MCAN region also contains many generalists, albeit a large majority of which are not as highly generalized as those seen in ENA. Twenty‐two of 59 species from the MCAN region are generalized, or 37.3% of MCAN natives.

### 3.4 Ancestral character state reconstruction

Across the *Quercus* phylogeny, there are some clades of highly generalized or specialized species (Figure 5). The overall pattern in the entire measured clade is one of the moderate generalization, with most ancestors beyond one node being estimated to have a ranking of ~44 (see Supporting Information‐Part 1 for additional details about the most generalized and specialized clades).

### 3.5 Mean metric‐based specialization rankings by ecoregion

Mean specialization ranks of ecoregions are significantly clustered (Moran's *I*, *p* < .0001; Figure 6), with a pattern of increasing specialization hotspots in regions at lower latitudes and high precipitation seasonality. Examples can be seen in the Southern Florida Coastal Plain, the US west coast, and regions spanning Central America to northern Mexico.

### 3.6 Specialization and climate

Metric‐based specialization scores and the mean precipitation seasonality by species were found to positively associated with no phylogenetic signal (*λ* = .04, *p* < .001, Figure 8). As seasonality increases, so do metric‐based rankings of specialization. On average, a species with a mean precipitation seasonality of 10 would likely have half the specialization score of a species with a seasonality of 120.

## 4 DISCUSSION

### 4.1 Viability of metric‐based ranking systems

Specialization in *Quercus* presents interesting insights into specialization as a whole. Creating a practical, objective ranking system of specialization is indeed possible. The ability to assess specialization in bulk could be of great use in studies involving a large number of related taxa (Catenazzi & von May, 2021; Mounce et al., 2018; Reece et al., 2013). Generated rankings provide a good basis of comparison for specialization, and extension threat level, which has utility for conservationists and scientists alike (Reece et al., 2013). Metric‐based rankings can be assumed to be accurate designations of specialization, given significant correlations to IUCN Red Listing designations and scientific assessment (Figure 3). Results indicate that experts are, on average, good at picking out specialized and generalized species, though they tend to rate species as slightly more specialized than a metric‐based system would (Figure 3). Correlation between survey responses and extent of occurrence indicate that experts may be using range sizes as a proxy for specialization.

Data‐deficient (DD) species tended to have higher specialization rankings. Of the 11 species with only two available metrics to be considered, two scored in the 70s, four in the 80s, and five in the 90s. Results also suggest some correlation between DD and threat level, so the omission of species lacking some metrics is not recommended; of the 11 species ranked highly with missing data, 36% are threatened on the Red List, including the only critically endangered species in this study, *Q*. *boyntonii*. Only 10.6% of the 141 species ranked were threatened. Deficiencies in data may be indicative of threat level for a variety of reasons (Todd & Burgman, 1998); species lacking information tend to be less widespread, understudied, and are potentially harder to access. Additionally, species relationships are underreported (McCann, 2007). Limited data on interactions between species may contribute to certain species' higher average ranking of specialization, and by extension, the higher threat level that we observed in data‐deficient species. A lack of interactions between species may not necessarily indicate higher or lower specialization; more data are needed to better explore this relationship.

Given the patterns regarding data deficiencies described above, our framework suggests that DD species should be prioritized for conservation, as they are more likely specialized compared to their well‐represented relatives. Results in Figure 3 also suggest that scientific literature containing specialists and generalist species (as decided by the authors) may be reliable regarding these designations, even if there is a lack of methodology or biological context provided. Results suggest some unity among scientific designation of specialization, and that there are potentially common characters experts are using in their designations, such as range size. In projects with few species, evaluating specialization could be accomplished through assessment from experts. However, a metric‐based approach may be advantageous when the study system gets increasingly large. Both approaches are potentially useful means of identifying threatened species (Figure 3).

### 4.2 Evolutionary and geographic patterns of specialization in Quercus

Specialization, and the metrics used herein to represent it, yield insightful phylogenetic and geographic trends. Metrics used to calculate specialization rankings, excepting of the number of interspecies interactions, tested as having phylogenetic signal (Table 4). Moderate K and *λ* values indicate a relatively low phylogenetic signal in specialization and its representative metrics. These results support the idea that the tested characters (besides DEL3) are more so emergent, rather than inherited features. Results suggest that while phylogenetic relationships play a role in the determination of specialization, species are being influenced by factors apart from relatedness (Li et al., 2016; Pearse & Hipp, 2012).

Interestingly, the number of distinct ecoregions a species inhabits (DEL3) shows the highest phylogenetic influence (*λ* = .83), indicative of high covariance in number of inhabited ecoregions, proportional to shared evolutionary history. Biodiversity richness patterns could be explained by differences in environmental heterogeneity within and among ENA and Mexico/Central America (Figures 6 and 7). The region a species occupies appears to drive overall specialization more so than phylogenetic relationships (sensu Guttová et al., 2019 and Manos, 2020). Specialization hotspots are clustered (Moran's *I*, *p* < .0001) and support our initial hypothesis that expanding generalist populations radiate into new heterogenous regions, and likely specialize into open niches similar to allopatric speciation.

Regions with high concentrations of specialists have increasingly extreme water availability (both more and less water) compared to ancestral distributions of North American oaks, namely those in ENA, as evidenced by increasing specialization from ENA to Mexico and Central America, and the positive correlation between precipitation seasonality and specialization ranking (Figures 6 and 8). Mexico, Central America, the US West Coast, and the US Southeast all tend to specialize the local oaks more heavily than ENA, likely due to a mix of harsh conditions that challenge more generalized species, namely the extremes of water availability (Ramírez‐Valiente et al., 2020). As shown in Figure 8, specialization is significantly correlated with high precipitation seasonality, with no phylogenetic signal. This indicates adaptation in response to environmental conditions, rather than phylogenetically influenced strategies.

Overall, specialization and generalization appear heavily controlled by the geographic region a species is native to, and as such, specialization tends to act as more of an emergent property of a place rather than a more typical inherited trait (Agrawal, 2019; Küttner et al., 2014). This is not too surprising, as specialization falls somewhere between an ecological strategy and a relative physiological state. It is useful to note, however, that there are differences between MCAN and ENA regarding ecoregions. Mexico is more heavily dissected at all ecoregion levels, while the differences in ENA are drastic between levels. This may have led to differential ranking potential for ENA species, as they have disproportionately more regions available as the ecoregion level increases. The MCAN region contains a relatively large number of species, and while many of them are highly specialized (36 species), this region also contains generalists (22 species). Appearance of generalists in MCAN could possibly be attributed to the fact that this region contains a great variety of environmental conditions driving speciation in an equally diverse manner.

Despite specialization appearing to be dictated largely by geographic influences, phylogenetic relationships have also played a part in specialization here and in other systems (Cooper & Lenski, 2000). Within clades and sections of *Quercus*, trends of specialization often tend to be preserved and clustered (Figure 4). This could be due sympatric species inhabiting similar environments, and therefore adopting similar strategies in response to those environments, rather than closely related species inheriting similar characteristics from ancestral populations (Hipp et al., 2018); however, phylogenetic overdispersion within communities may run counter to this pattern (Cavender‐Bares et al., 2018). Coexistence between specialists and generalists within a group can be restricted (Egas et al., 2004), which may help to explain species whose metric‐based specialization rankings are contrary to their sister taxa.

Overall, *Quercus* species tend to come from generalized ancestors (Figure 5). Results of Ancestral Character State Reconstruction suggest that clades of highly generalized or specialized species arise from ancestral oak populations that maintain a moderate level of generalization. Species ranked contrary to their close relatives may have undergone allopatric speciation across varied regions, resulting in environmental pressures that selected for a different strategy (Aguilar‐Romero et al., 2017). This may explain large cases of variation in specialization rankings across regions, such as those seen in section Lobatae and section Quercus.

Utilization of this metric‐based process could easily be adapted to other systems and may yield useful insights into how specialization emerges, and the evolutionary timescale at which it does so. Additional geographic and biological research should also be directed into how water stress seasonality (i.e., BIO15) may drive specialization and what may occur to endemic species under rapid climate change (Hanson & Weltzin, 2000).

## AUTHOR CONTRIBUTIONS

**Alex Nicholas Kirsch:** Conceptualization (equal); data curation (equal); formal analysis (equal); funding acquisition (supporting); investigation (equal); methodology (equal); project administration (supporting); resources (supporting); software (equal); supervision (supporting); validation (supporting); visualization (equal); writing – original draft (lead); writing – review and editing (equal). **Matthew A Kaproth:** Conceptualization (equal); data curation (equal); formal analysis (equal); funding acquisition (lead); investigation (equal); methodology (equal); project administration (lead); resources (lead); software (equal); supervision (lead); validation (lead); visualization (equal); writing – original draft (supporting); writing – review and editing (equal).

## CONFLICTS OF INTEREST

The authors have no conflicts of interest to disclose.

### OPEN RESEARCH BADGES

This article has earned an Open Data badge for making publicly available the digitally‐shareable data necessary to reproduce the reported results. The data is available at https://github.com/mkaproth/QuercusSpecialization.

## Supporting information


Appendix S1.

## Data Availability

Data available through GitHub at https://github.com/mkaproth/QuercusSpecialization.
