# Supplementary material for: Defining plant ecological specialists and generalists: Building a framework for identification and classification
Source: Ecol Evol. 2022 Nov 24;12(11):e9527. doi: 10.1002/ece3.9527 (PMC9685674; doi:10.1002/ece3.9527)
Supplement: Supplementary file 1 — Appendix S1. [file ECE3-12-e9527-s001.docx]

**Supporting Information - Part One**

*Survey Sample*

Survey respondents were asked both to define ecological specialization and generalization, and to rank individual species on their level of ecological specialization. Questions one and two asked respondents “How would you define a plant that is an ecological specialist?” and “How would you define a plant that is an ecological generalist?”. All remaining questions asked respondents to rank a species based on its level of ecological specialization, with a five being the most specialized an oak can be and a one being the most generalized (these directions were provided at the head of the survey). Each question also provided a link to the corresponding species’ page on the Oaks of the World website, so that they could clarify which species they were being asked about, as some species have been subject to taxonomic changes. An example is provided below.

Example Question:

*Quercus lyrata*

*http://oaks.of.the.world.free.fr/quercus_lyrata.htm*

- 5
- 4
- 3
- 2
- 1
- No Familiarity


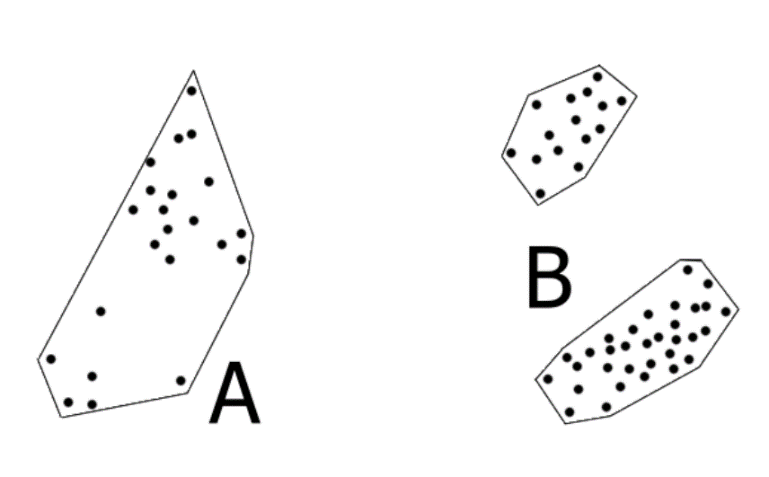


***Supporting Information Fig. S1****.* Examples of how Extent of Occurrence was drawn and calculated in ArcMap for two species, A and B. Using multiple polygons for a species was reserved for only a small number of instances where the space between polygons was more than likely uninhabitable for that species, as was the case with some Mexican taxa native to high altitude mountain ranges.

*Explanation of* ***Formula 1*** *(Percentile Scoring) and Example*

***Formula 1****.*

1. $(Species EOO/ 957,618 km^{2})$ = A
2. 1 - A = B
3. B * 25 = # Points Assigned

Step one of formula 1 represents where a species falls in the context of all EOO values (957,618km² is the highest EOO before the 20^th^ percentile cutoff, and is used as the max range). Step two inverts this, since higher EOO’s represent lower specialization. In step 3, the value from step 2 is multiplied by total available points for this metric (25), resulting in the number of points the species gets for this metric. An example is provided below using *Quercus lancifolia (EOO = 343,905km²)*.

1. 342,905km²/957,618km² = 0.359
2. 1 – 0.359 = 0.641
3. 0.641 * 25 = 16.02 Points awarded for EOO

*Plasticity Traits and* ***Formula 2***

Functional traits measured and used for plasticity were Petiole Length, Leaf Length, Leaf Lobedness, and Specific Leaf Area (Cornelissen et al. 2003). These 4 metrics were chosen due to both model selection, and their impact on an individual’s ability to inhabit certain regions. They were not the only traits measured, however (*Table 3*). Minimums and maximums of these traits across individuals of a given species were used in ***Formula 2*** to calculate plasticity.

***Formula 2***

$$\frac{\left( Max of Trait-Min of Trait \right)}{(Max of Trait+Min of Trait)}$$

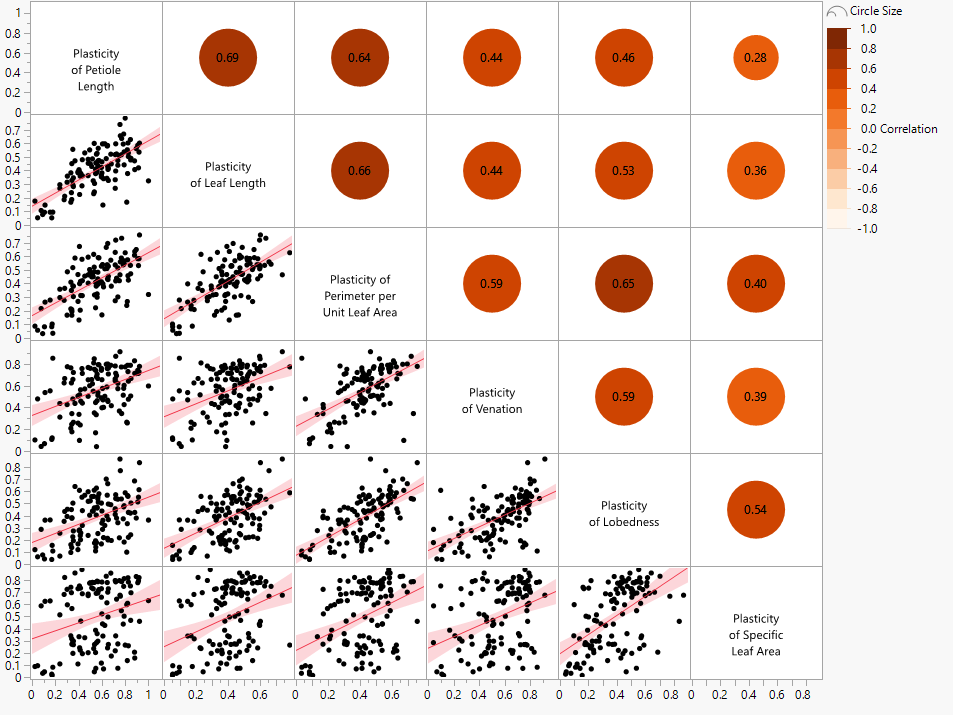


***Supporting Information Fig. S2****.* Regression Matrix showing correlations between leaf functional traits (Generated in JMP version 15.1.0, Analyze, Multivariate). Leaf functional traits show a high degree of correlation between one another. Species with a high or low plasticity for one leaf trait tend to exhibit a similar state for all of them. With a mean correlation coefficient of 0.55, plasticity in functional leaf traits is positively correlated for every combination of the six traits collected for this study. AICc model selection retained four of the six available functional leaf traits when predicting IUCN Red List designation and the average response score from the specialization survey (*Table 3*), suggesting that including multiple measures of plasticity in similar studies may bolster predictive power. As a trait thought to be tied to generalization, metrics of plasticity have additional value in representing this concept apart from improving predictive models.

*Specialization and Generalization across* Quercus *clades*

The most generalized groups are seen in Eastern North American (ENA) species, such as the species from *Q. prinoides* through *Q. alba* (*Quercus* subsections *Prinoideae and Albae* (Trel.) A. Camus), and *Q. palustris* though *Q. laurifolia* (*Quercus* subsections *Phellos, Coccineae,* and *Palustres*). The most specialized species are almost exclusively natives of Mexico, Central America, Arizona and New Mexico (MCAN), such as *Q. laceyi* (rank of 77.1)*,* *Q. mohriana* (rank of 66.1), and *Q. canbyi* through *Q. uxoris* (ranks from 61.8 to 81.9, Lobatae and *Quercus* subsection Erythromexicana). Some highly specialized species that are not native to the MCAN region include *Q. myrtifolia*, an ENA native (rank of 74.3), and *Q. cornelius-mulleri* through *Q. pacifica* (*Quercus* subsects *Dumosae* and *Prinoideae*), natives of the California Floristic Province and the Pacific Northwest (CFPN) (ranks from 62.8 to 69.8).

*Global Biotic Interactions (GloBI)*

GloBI is an open access search tool/conglomerate database that contains 10,006,690 (7,077,559) interaction records that span 727,371 taxa as of November 6^th^, 2021. These interactions can be sorted by the type of interaction, and by taxa the user is interested in. Records were filtered for each species, with types of interactions not relevant to *Quercus* omitted. The included interaction types were the following:

Commensalist, Dispersal Vector, Ecologically Related to, Flowers Visited by, Mutualist of, Pollinator, and Symbiont.

The number of known interactions for each species was recorded, and were used with ***Formula 1***. The inversion step of ***Formula 1*** was *not* used for this factor, as more specialized interactions are assumed to reflect higher specialization.

*Domatia*

Anatomical features with narrow uses are a key aspect of specialized species. Although *Quercus* is considered largely generalist at a broader biological level, domatia represent specialized anatomy that can be assessed for the oaks. In *Quercus,* domatia are small chambers made of trichomes at the intersections along the mid-vein of the leaf. These are created to shelter beneficial arthropods that likely help reduce herbivory on the tree. Presence or absence of domatia may be interpreted as being indicative of interspecies specialization.

Domatia presence or absence was assessed for three individuals per species. Each of the three samples was denoted with a 0 (no domatia), 1 (hair present but likely non-functional), or 2 (functional domatia present). These were summed per species, and the totals were scored using ***Formula 1***, minus the inversion, as a higher domatia presence is interpreted as higher specialization.

***Supporting Information Table 1****.* Blomberg’s K and Pagel’s Lambda for Plasticity of Individual Leaf Traits using mean values per species across *Quercus* phylogeny

| **Plasticity of Leaf Trait** | ***K*** | **p** | **λ** | **p** |
| --- | --- | --- | --- | --- |
| Petiole Length | 0.103068 | 0.857 | 0.0000681 | 1 |
| Leaf Length | 0.118428 | 0.4 | 0.0000681 | 1 |
| Perimeter per unit Leaf Area | 0.11096 | 0.637 | 0.0000681 | 1 |
| Venation | 0.102882 | 0.875 | 0.0000681 | 1 |
| Leaf Lobedness | 0.103159 | 0.863 | 0.0000681 | 1 |
| Specific Leaf Area | 0.11603 | 0.464 | 0.0000681 | 1 |


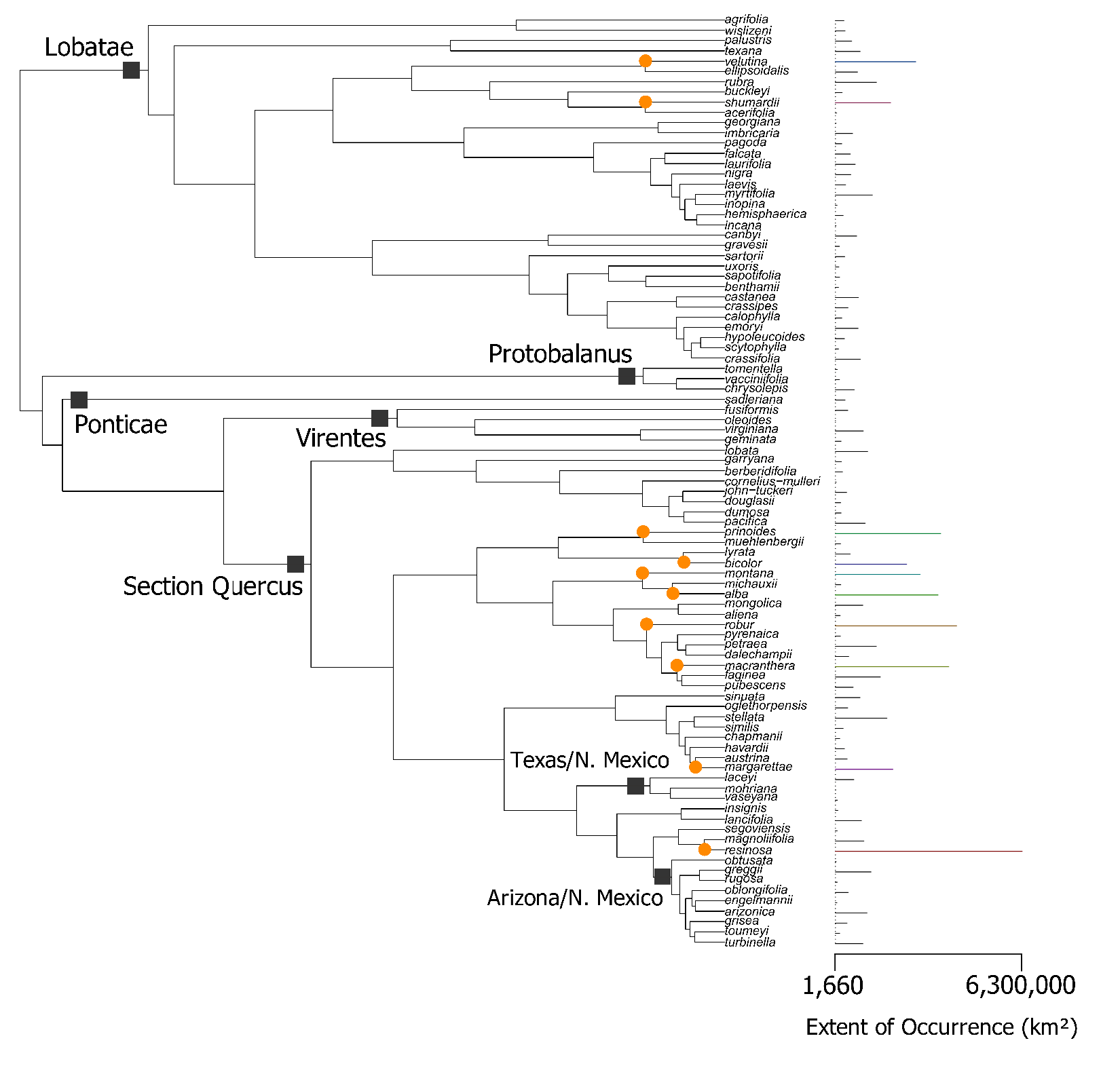


***Supporting Information Fig. S3****.* Phylogenetic EM results for Species Extent of Occurrence. EOO (Km²) was the only trait of those considered for specialization that showed phylogenetic shifts. Shifts are represented by the orange circles.


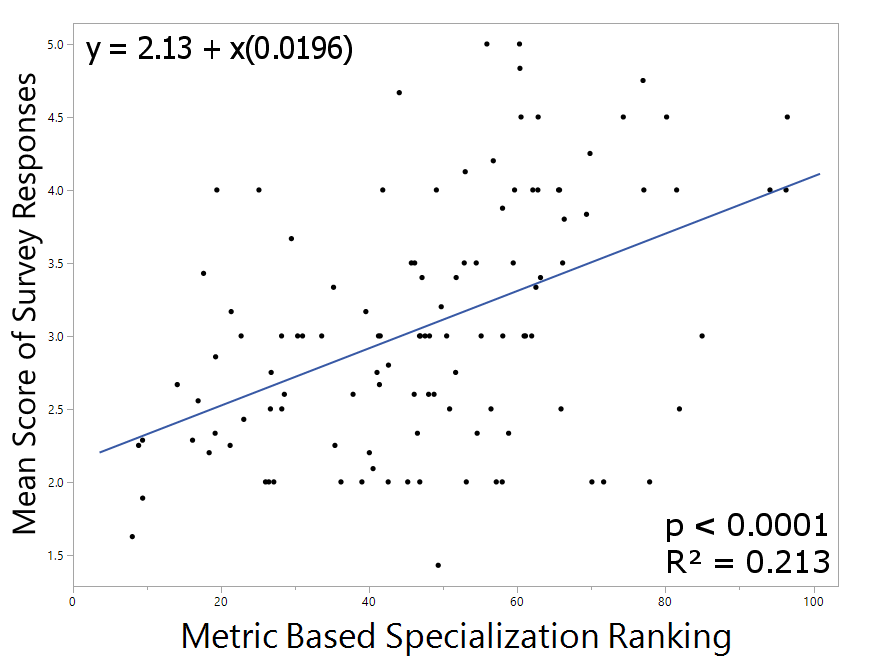


***Supporting Information Fig. S4.***  Correlation between mean score of survey responses and Metric-Based Specialization Rankings, displayed with p, R², and the equation of the line of best fit.


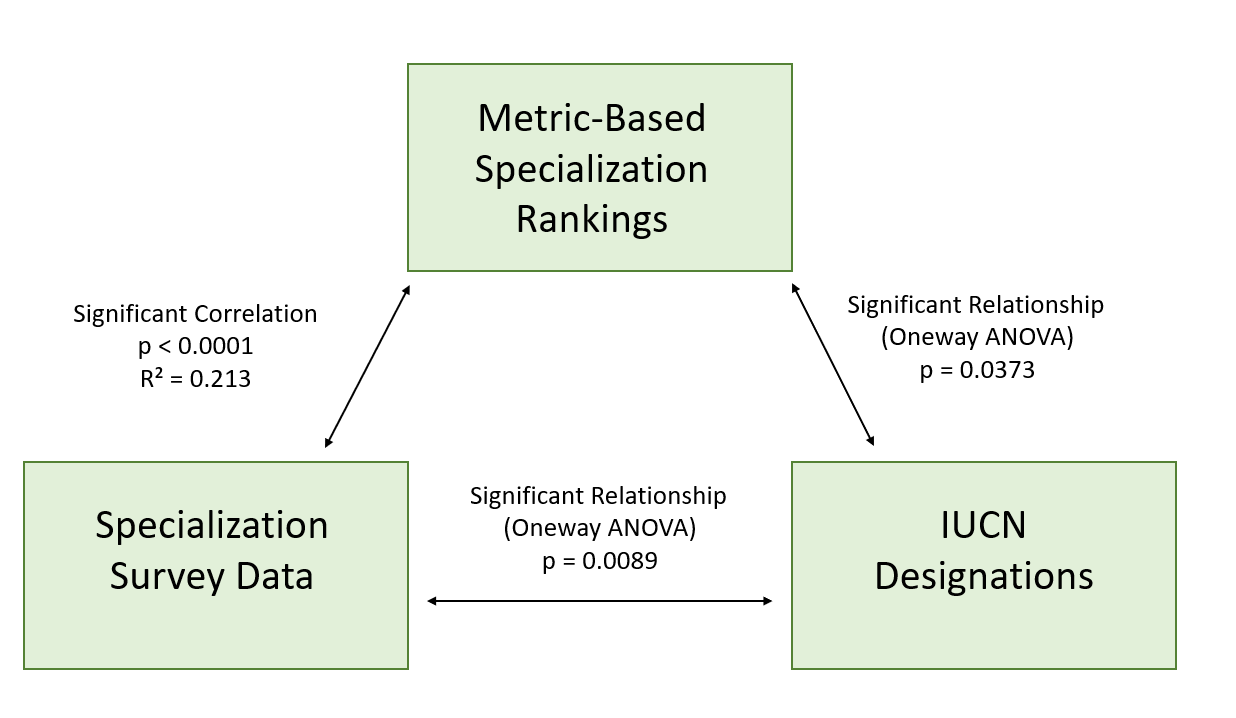


***Supporting Information Fig. S5****.* Results of statistical analysis between Specialization Rankings, results of the specialization survey, and IUCN designations. Significance levels and the type of test performed are shown by the double-sided arrows between data. All groups significantly tested against the other two.

**Supporting Information - Part Two: Data and Coding Scripts**

***Supporting Information Table 2****. Subset of Quercus species ranking data used to parameterize metric generalist-specialist rankings (pre-model selection).*

| Species | acerifolia | calophylla | lancifolia | mohriana | uxoris |
| --- | --- | --- | --- | --- | --- |
| Native Region (Main) | E | M | M | M | M |
| Native Region (Sub) |  |  |  | E |  |
| Extent of Occurrence (km²) | 38725.81 | 715975.09 | 343905.79 | 433384.5 | 114800.89 |
| Distinct Inhabited Ecoregions at Level III | 3 | 20 | 12 | 11 | 4 |
| Distinct Inhabited Ecoregions at Level II | 2 | 17 | 9 | 7 | 3 |
| Distinct Inhabited Ecoregions at Level I | 1 | 8 | 4 | 4 | 2 |
| Domatia Score Total | 4 | 5 | 7 | 3 | 5 |
| Number of Notable, Documented Interspecies Interactions (GloBi) | 0 | 0 | 2 | 0 | 0 |
| Plasticity of Petiole Length | 0.30300807 | 0.178082192 | 0.625 | 0.574468 | 0.536679537 |
| Plasticity of Leaf Length | 0.359649123 | 0.095477387 | 0.597444089 | 0.474747 | 0.305882353 |
| Plasticity of Perimeter per Unit Leaf Area | 0.486989577 | 0.035800454 | 0.508829966 | 0.589311 | 0.317107169 |
| Plasticity of Venation | 0.774674719 | 0.85223613 | 0.503628138 | 0.792529 | 0.751401532 |
| Plasticity of Leaf Lobedness | 0.453223543 | 0.108213589 | 0.319516002 | 0.581022 | 0.174577189 |
| Plasticity of Specific Leaf Area | 0.597289489 | 0.081812091 | 0.427190396 | 0.723584 | 0.269431712 |
| Metric-Based Specialization Rank (AICc) | 60.33916445 | 28.15050487 | 41.81753187 | 66.10377 | 61.08799708 |
| Average Score from Expert Survey | 4.833333333 | 3 | 4 | 3.5 | 3 |
| IUCN Status | EN | NT | LC | LC | LC |
| IUCN Status (Threatened, Near Threatened, Least Concern) | THR | NT | LC | LC | LC |

***Plotting Metric-Based Specialization Rankings as Color-Coded Bars at Tips of Phylogeny (R Script)***

#Set Working Directory to Location of Data Files

setwd("C:/Users/Alex/Desktop/R")

###LIBRARIES###

library(phytools)

library(magrittr)

###READING IN DATA###

#Read in Metric Data from Excel (.csv)

traits <- read.csv("MainMetrics3821.csv", as.is=TRUE)

#Read in List of Species with Data Deficiencies

NADropList <- read.csv("NADropList.csv", as.is=TRUE)

#Read in the primary phylogenetic tree

tr <- read.tree("tr.singletons.GlobalOaks2019.tre")

#strip genera from each species binomial

tr$tip.label <- gsub('Quercus_', '', tr$tip.label, fixed = T)

#make a list of species in .tre

tr$tip.label <- sapply(strsplit(tr$tip.label, "|", fixed = T),

'[', 1) %>% make.unique

#Create tr.pruned; tree with only species in Specialization Study

tr.pruned <- drop.tip(tr, which(!tr$tip.label %in% traits$Species))

#Changes row numbers to species names

traits <- read.csv("MainMetrics3821.csv", as.is=TRUE, row.names = 1)

###PLOTTING###

#The lines below plot Metric-Based Specialization Rankings as color coded bars

#at the respective tips of the tree. The object 'Colors' is a vector of colors

#telling the plotting function what color each species' bar should be, that

#must be declared prior to plotting. Dashed lines and the text labels denoting

#them were added with the 'text' and 'abline' functions. View at 20 x 20.

#Creates object 'Ranks', a named vector of Metric-Based Rankings.

Ranks<-setNames(traits$RankAICc,rownames(traits))

#Plots a tree with bars, where the bars are scaled by Metric-Based Ranking

#and color coded by native region.

plotTree.wBars(tr.pruned, Ranks, col= Colors, tip.labels=TRUE)

***Ancestral Character State Reconstruction (R Script)***

#Set Working Directory to Location of Data Files

setwd("C:/Users/Alhex/Desktop/R")

###LIBRARIES###

library(ape)

library(magrittr)

library(nlme)

library(phytools)

library(tidyverse)

###READING IN DATA###

#Read in Metric Data from Excel (.csv)

traits <- read.csv("MainMetrics3821.csv", as.is=TRUE)

#Read in List of Species with Data Deficiencies

NADropList <- read.csv("NADropList.csv", as.is=TRUE)

#Read in the primary phylogenetic tree

tr <- read.tree("tr.singletons.GlobalOaks2019.tre")

#strip genera from each species binomial

tr$tip.label <- gsub('Quercus_', '', tr$tip.label, fixed = T)

#make a list of species in .tre

tr$tip.label <- sapply(strsplit(tr$tip.label, "|", fixed = T),

'[', 1) %>% make.unique

#Creates tr.nonafinal; tree with only species that have no data deficiencies

tr.nonafinal <- drop.tip(tr, which(!tr$tip.label %in% NADropList$Species))

#Changes row numbers of Metric Data table to species names

traits <- read.csv("MainMetrics3821.csv", as.is=TRUE, row.names = 1)

###ESTIMATING AND PLOTTING ANCESTRAL STATES

#Creates vector of species names on the tree

nonafinaltips <- tr.nonafinal[["tip.label"]]

#Create object of Metric-Based Rankings of Specialization, 'SpecRanks'

SpecRanks <- traits[nonafinaltips, 27]

#Assign species names to SpecRanks

names(SpecRanks) <- nonafinaltips

#Changes Data to Correct Input Format

SpecRanksDF <- as.data.frame(SpecRanks, row.names = nonafinaltips)

SpecRanksMTX <-as.matrix(SpecRanksDF)

SpecRanksFinal<-as.matrix(SpecRanksMTX)[,1]

#Perform the Ancestral State Estimations

AncestralStates <- fastAnc(tr.nonafinal, SpecRanksFinal, vars=FALSE,CI=FALSE)

#This Plots Ancestral States as a colored gradient across branches

#View at 17x17 Window size

cont<-contMap(tr.nonafinal,SpecRanksFinal,plot=FALSE)

plot(cont,legend=0.7*max(nodeHeights(tr.nonafinal)), mar=c(5,5,5,5))

#Plots the Tree by Current and Estimated States as a phenogram across time

#View at 15x15 window size

par(mar=c(5,4,4,5))

phenogram(tr.nonafinal,SpecRanksFinal,

spread.labels=TRUE,spread.cost=c(1,0),

link=2.5 , offset=0)

#This Plots the Plain Tree with Nodes Labeled by Circles,

#Scaled by Estimated State

#View at 15x15 window size

plot(tr.nonafinal)

nodelabels(pch = 21, cex=(AncestralStates/20),

bg=ifelse(AncestralStates>50,"Black","White"))

***Phylogenetic Generalized Least Squares (R Script)***

#Set Working Directory to Location of Data Files

setwd("C:/Users/Alhex/Desktop/R")

###LIBRARIES###

library(ape)

library(magrittr)

library(nlme)

###READING IN DATA###

#Read in Data from Excel (.csv)

traits <- read.csv("WaterPGLS.csv", as.is=TRUE)

#Read in the primary phylogenetic tree

tr <- read.tree("tr.singletons.GlobalOaks2019.tre")

#Strip genera from each species binomial

tr$tip.label <- gsub('Quercus_', '', tr$tip.label, fixed = T)

#Make a list of species in .tre

tr$tip.label <- sapply(strsplit(tr$tip.label, "|", fixed = T),

'[', 1) %>% make.unique

#Create tr.pruned; tree with only species in Specialization Study

tr.pruned <- drop.tip(tr, which(!tr$tip.label %in% traits$Species))

#Changes row numbers to species names

traits <- read.csv("WaterPGLS.csv", as.is=TRUE, row.names = 1)

#creates vector of species name

tips <- tr.pruned[["tip.label"]]

###PREPPING VARIABLES###

#Save metrics to be tested as vectors, then name them with the respective

#species names.

Rank <- traits[tips, 3]

IMGS <- traits[tips, 1]

Bio15 <- traits[tips, 2]

names(Rank) <- (tips)

names(IMGS) <- (tips)

names(Bio15) <- (tips)

###RUN TEST AND VIEW TEST RESULTS###

#PGLS model of Metric-Based Rank against Bioclimatic Variable 15

pglsModel1 <- gls( Rank ~ Bio15,

correlation = corPagel(value = 1, phy = tr.pruned, fixed = FALSE, form = ~1),

data = traits, method = "ML")

#Display test summary and coefficient

summary(pglsModel1)

coef(pglsModel1)
